# Differences in Gut Microbiome Composition Between Sympatric Wild and Allopatric Laboratory Populations of Omnivorous Cockroaches

**DOI:** 10.3389/fmicb.2021.703785

**Published:** 2021-07-28

**Authors:** Kara A. Tinker, Elizabeth A. Ottesen

**Affiliations:** Department of Microbiology, University of Georgia, Athens, GA, United States

**Keywords:** cockroach, gut microbiome, host–microbe, 16S rRNA amplicon sequencing, sympatric

## Abstract

Gut microbiome composition is determined by a complex interplay of host genetics, founder’s effects, and host environment. We are using omnivorous cockroaches as a model to disentangle the relative contribution of these factors. Cockroaches are a useful model for host–gut microbiome interactions due to their rich hindgut microbial community, omnivorous diet, and gregarious lifestyle. In this study, we used 16S rRNA sequencing to compare the gut microbial community of allopatric laboratory populations of *Periplaneta americana* as well as sympatric, wild-caught populations of *P. americana* and *Periplaneta fuliginosa*, before and after a 14 day period of acclimatization to a common laboratory environment. Our results showed that the gut microbiome of cockroaches differed by both species and rearing environment. The gut microbiome from the sympatric population of wild-captured cockroaches showed strong separation based on host species. Laboratory-reared and wild-captured cockroaches from the same species also exhibited distinct gut microbiome profiles. Each group of cockroaches had a unique signature of differentially abundant uncharacterized taxa still present after laboratory cultivation. Transition to the laboratory environment resulted in decreased microbiome diversity for both species of wild-caught insects. Interestingly, although laboratory cultivation resulted in similar losses of microbial diversity for both species, it did not cause the gut microbiome of those species to become substantially more similar. These results demonstrate how competing factors impact the gut microbiome and highlight the need for a greater understanding of host–microbiome interactions.

## Introduction

The gut microbiome plays an important role in the health and fitness of most animals. Gut microorganisms assist with the breakdown of dietary substrates and play a role in nutritional absorption and energy regulation (Dillon and Dillon, 2003; Engel and Moran, 2013). The gut microbiome also protects against pathogens, both by inhibiting colonization by invading pathogenic microbes as well as by interacting with the host immune (Engel and Moran, 2013). Certain toxins, including pesticides, can be metabolized by the gut microbiome (Engel and Moran, 2013; Brune and Dietrich, 2015; Claus, 2016). The presence of certain gut microbes is essential for the development of several types of insects, including the cockroach and mosquito (Bracke et al., 1978; Cruden and Markovetz, 1984; Gijzen and Barugahare, 1992; Coon et al., 2015; Jahnes and Sabree, 2020). Finally, recent work demonstrates that microbes can impact animal behavior across classes, from insects to mammals (Wada-Katsumata et al., 2015; Vuong et al., 2017). Examples include frequency of social interactions, mate choice, hyperactivity, anxiety, depression, and others (Wada-Katsumata et al., 2015; Delbare et al., 2020; Heys et al., 2020; Zhu et al., 2020). Therefore, identifying and understanding host–gut microbiome interactions is an important area of research.

Gut microbiome composition is determined by a complex interplay of host genetics, early environment, and immediate environment (Turnbaugh et al., 2009; Neu and Rushing, 2011; Ericsson and Franklin, 2015; Martinson et al., 2017; Ho et al., 2018; Kakumanu et al., 2018; Lee et al., 2020; Lim and Bordenstein, 2020; Sepulveda and Moeller, 2020; Tinker and Ottesen, 2020; Wolff et al., 2020). Host genetics has been shown to play the key role in the gut microbiome of certain species, including cockroaches, mosquitoes, and apes (Sanders et al., 2014; Novakova et al., 2017; Lim and Bordenstein, 2020; Tinker and Ottesen, 2020). In contrast, sympatric populations of *Drosophila* (Martinson et al., 2017) were shown to exhibit indistinguishable gut microbial communities, suggesting environment may be the primary factor in shaping the gut microbiome for certain species. Early environment and founder’s effects are also thought to play a role in the gut microbiome, with studies in humans showing lasting signatures arising from mode of birth (vaginal vs. cesarean) and newborn diet (breastfeeding vs. formula feeding) (Neu and Rushing, 2011; Ho et al., 2018). Additionally, recent work suggests that the host’s immediate environment can cause major impacts on the gut microbiome. For instance, temperature (Sepulveda and Moeller, 2020), diet (Turnbaugh et al., 2009), and/or housing conditions (isolated vs. co-housed) (Ericsson and Franklin, 2015; Caruso et al., 2019) can be manipulated in order to produce certain gut microbiome profiles in mice.

Cockroaches are an ideal organism for studying gut microbial community assembly and host–microbe interactions. Cockroaches represent an ancient lineage that emerged over 300 million years ago (Legendre et al., 2015; Wang et al., 2017) and have evolved to host a complex gut microbiome composed of hundreds of unique species of microbes (Bell et al., 2007; Engel and Moran, 2013; Dietrich et al., 2014). This gut microbiome is composed of insect-associated lineages, but is populated by microbial families and genera that are characteristic of the gut microbiomes of a wide range of omnivorous animals, including mice, and humans (Schauer et al., 2012; Dietrich et al., 2014; Tinker and Ottesen, 2016). The cockroach gut microbial community is not vertically transmitted, but indirectly acquired from other cockroaches in their immediate environment, a process aided by their typically gregarious lifestyle (Bell et al., 2007; Engel and Moran, 2013; Wada-Katsumata et al., 2015; Kakumanu et al., 2018). Acquisition of a healthy gut microbial community is required for proper development (Bracke et al., 1978; Cruden and Markovetz, 1984; Gijzen and Barugahare, 1992; Jahnes and Sabree, 2020). Dysbiotic cockroaches are generally smaller than their healthy counterparts and rarely complete the final molt into adulthood (Bracke et al., 1978; Cruden and Markovetz, 1984; Gijzen and Barugahare, 1992; Jahnes and Sabree, 2020). Social behavior is also impacted by the gut microbiome, which produces volatile carboxylic acids (VCAs) which act as aggregation agents for the insect hosts (Wada-Katsumata et al., 2015). Axenic cockroaches produce no aggregation agents and thus have reduced social contact from other insects (Wada-Katsumata et al., 2015). Interestingly, once assembled, the cockroach gut microbiome appears to be robust to many environmental perturbations (Renelies-Hamilton et al., 2021). This may be due to regular conspecific inoculation events through social behaviors including coprophagy and trophallaxis (Renelies-Hamilton et al., 2021).

In this experiment, we used 16S rRNA gene sequencing to compare the hindgut microbiome of an in-house laboratory colony of *Periplaneta americana*, a second laboratory colony of *P. americana* obtained from the University of Florida, and sympatric wild-caught *P. americana* and *Periplaneta fuliginosa*. A subset of the transplanted laboratory colony and wild-caught cockroach populations were maintained under in-house laboratory conditions for a period of 14 days to observe changes in the hindgut microbiome following this transition. We were specifically interested in measuring changes in richness (alpha diversity) and dissimilarity (beta diversity) within and across the treatment groups after a transition into a laboratory environment. We were also interested in identifying which factors (treatment group, host species, and/or sample origin) most impacted the hindgut microbial community. Finally, we planned to identify and investigate any taxa that were uniquely associated with particular treatment groups.

## Materials and Methods

### Insects

Our in-house laboratory colony of *P. americana* cockroaches was provided by the University of Georgia’s entomology department from a colony that has been maintained in captivity for over 10 years. The cockroaches were maintained in mixed-age, mixed-sex colonies in aquarium tanks at room temperature on a diet of dog food (Kroger nutritionally complete bite-sized adult dog food, composed of 21% protein, 8% fat, and 6% fiber) *ad libitum*. Each tank was provided with corn cob bedding, cardboard tubes for nesting, and a cellulose sponge saturated with water. The laboratory colony from the University of Florida was generously obtained and provided by Brian Forschler, a colleague in the University of Georgia’s entomology department.

For studies of wild-caught cockroaches, insects were collected in traps placed outside within a 135 m radius on the University of Georgia’s campus. The traps were glass jars with petroleum jelly placed around the jar opening to prevent insects from escaping. Each trap contained glass wool saturated with beer as a lure. Traps were checked daily, and any captured *P. americana* and *P. fuliginosa* adults were either sacrificed immediately or placed in an aquarium tank under laboratory culture conditions (as described above) for 14 days before being sacrificed. Wild *P. americana* and *P. fuliginosa* were visually identified by morphology. We confirmed our identifications by sequencing a representative sample from each insect species. For the *P. americana* sample we sequenced a modified A-tLeu/B-tLys and for the *P. fuliginosa* sample we sequenced the CO-II gene, both as previously described (Tinker and Ottesen, 2020). Ultimately, our experiment included a total number of 90 insect samples from the following groups: laboratory *P. americana* (12 samples), Florida laboratory *P. americana* at time 0 (8 samples), Florida laboratory *P. americana* at day 14 (20 samples), wild *P. americana* at time 0 (12 samples), wild *P. americana* at day 14 (11 samples), wild *P. fuliginosa* at time 0 (12 samples), and wild *P. fuliginosa* at day 14 (15 samples) (Supplementary Table 1). Each sample represented an individual dissected hindgut.

### Hindgut Collection and DNA Extraction

We opted to focus on the hindgut in this study due to its high bacterial density and diversity (Cruden and Markovetz, 1987). For this work, DNA was extracted from individual dissected hindguts; no hindguts were pooled. Each insect was placed on ice in a sterile culture plate until sufficiently torpid. The entire cockroach gut was dissected and any visible debris, including fat bodies or exoskeleton, was removed with forceps. The hindgut was then separated from the rest of the gut, placed on parafilm, and submerged in 100 μL of RNAlater (Ambion, Austin, TX, United States). A pipette tip was used to break open the hindgut and disperse the contents into the RNAlater (Ambion) before the suspended hindgut lumen was removed and stored at 80°C.

DNA was extracted from a 30 μL aliquot of the preserved hindgut sample using a modified version of the EZNA Bacteria kit (Omega Biotek, Norcross, GA, United States). A 100 μL of balanced salt solution (2.5 g K2HPO4, 1 g KH2PO4, 1.6 g KCl, 1.4 g NaCl, and 10 ml of 1 M NaHCO3 per liter, pH 7.2) was added to each sample aliquot before mixing followed by centrifugation for 10 min at 5,000 × *g*. The supernatant was discarded and the pellet was resuspended in 100 μL TE buffer [10 nM Tris, 1 mM EDTA (pH 8)] with 10 μL lysozyme (as supplied by kit). The sample was incubated at 37°C for 30 min before adding approximately 25 mg of glass beads (as supplied by kit) and bead beating for 5 min at 3,000 rpm. Hundred microliters BTL buffer and 20 μL proteinase K solution (as supplied by the kit) were added to each sample before incubation at 55°C while shaking at 600 rpm for 1 h. After this step, the manufacturer’s protocol (June 2014 version) was followed beginning at step 11. Samples were eluted in 50 μL preheated elution buffer after a 5-min incubation at 65°C. The final DNA concentrations (typically between 5 and 50 ng/μL) and A260/A280 were measured using a NanoDrop Lite spectrophotometer (Thermo Scientific, Wilmington, DE, United States).

### Library Preparation and Sequencing

The V4 region of the 16S rRNA gene from each hindgut sample was amplified using a two-step PCR method as previously described (Tinker and Ottesen, 2016, 2018, 2020; Hassell et al., 2018). In brief, the initial PCR used Q5 Hot Start high-fidelity DNA polymerase (New England BioLabs, Ipswich, MA, United States) and 515F (GTGCCAGCMGCCGCGGTAA) and 806R (GGACTACHVGGGTWTCTAAT) primers in a 10 μL PCR mixture [1 Q5 reaction buffer, 200 M deoxynucleoside triphosphates (dNTPs), 0.5 M 515F, 0.5 M 806R, 2 ng DNA, and 0.02 U/L Q5 polymerase] under the following conditions: 98°C for 30 s, followed by 15 cycles at 98°C for 10 s, 52°C for 30 s, and 72°C for 30 s, with a final extension step at 72°C for 2 min for the initial V4 region amplification. Immediately following the initial amplification, the resulting product was reamplified using double barcode primers (Tinker and Ottesen, 2016, 2018, [Bibr B53][Bibr B19]). The secondary amplification mixture contained 1 Q5 reaction buffer, 200 M dNTPs, 0.5 M 515F, 0.5 M 806R, 2 ng DNA, and 0.02 U/L Q5 polymerase. From this mixture, 21 μL was added to 9 μL of the initial reaction product before cycling under the following conditions: 98°C for 30 s, followed by four cycles at 98°C for 10 s, 52°C for 10 s, and 72°C for 30 s, followed by six cycles at 98°C for 10 s and 72°C for 1 min, concluding with a final extension at 72°C for 2 min. The resulting PCR amplicons were purified using the EZNA Cycle Pure kit (Omega Bio-tek) before quantification with a NanoDrop Lite spectrophotometer (Thermo Scientific). Amplicons were then normalized to equal concentrations, pooled to a library concentration of 10 nM, and assessed for quality using the Agilent 2100 Bioanalyzer DNA-HS assay (Agilent Technologies, Santa Clara, CA, United States) before submission to the Georgia Genomics Facility for sequencing (Illumina MiSeq 250 × 250 bp; Illumina Inc., San Diego, CA, United States).

### Data Analysis

16S rRNA gene sequences were analyzed using a modified version of the Mothur Miseq standard operating protocol (Schloss et al., 2009). In brief, after sequence assembly any sequence that had ambiguous bases or was longer than 275 bp was removed and remaining sequences were aligned to the Silva reference database (Release 132) (Pruesse et al., 2007; Quast et al., 2012). Aligned sequences that contained homopolymers of 8 or more base pairs were removed before chimeral identification and removal via VSEARCH (Rognes et al., 2016). Remaining sequences were classified using the Silva reference database (Release 138) (Pruesse et al., 2007; Quast et al., 2012). Unclassified sequences or sequences identified as chloroplasts, mitochondria, Eukaryota, or *Blattabacterium* (cockroach endosymbiont found in fat body cells) were removed. The remaining sequences were clustered into OTUs based on 97% or greater sequence identity using OptiClust (Westcott and Schloss, 2017).

Data generated by Mothur was imported into R for further analysis (Schloss et al., 2009). The vegan (Oksanen et al., 2012) package was used to complete non-metric multidimensional scaling (NMDS) analyses and Analysis of Similarities (ANOSIM). Vegan (Oksanen et al., 2012) was also utilized to calculate Shannon diversity and Bray–Curtis dissimilarity values. Wilcoxon rank-sum tests were run using base R and differential abundance was measured with the DESeq2 (Love et al., 2014) package. Finally, we used base R, ggplot (Wickham, 2016), and RColorBrewer (Neuwirth, 2014) to visualize the data.

### Data Mining

Our analysis utilizes previously published data as well as new, unpublished data. Please note that the 16S rRNA sequencing data from the laboratory *P. americana* (Tinker and Ottesen, 2016), wild *P. americana* at both timepoints (Tinker and Ottesen, 2016), and wild *P. fuliginosa* at time 0 (Tinker and Ottesen, 2020) were utilized in previous publications. These publications investigated the effect of diet on *P. americana* (Tinker and Ottesen, 2016) and measured the phylosymbiotic signature in omnivorous cockroaches (Tinker and Ottesen, 2020). The 16S rRNA sequencing data from the wild *P. fuliginosa* at day 14 as well as the laboratory Florida *P. americana* at both time points is new, previously unpublished data. The methods described above were used to generate all data, both published and unpublished.

## Results

### All Cockroaches Harbor a Hindgut Microbiome Dominated by Members of the Bacteroidota, Firmicutes, and Desulfobacterota Phyla

**FIGURE 1:**
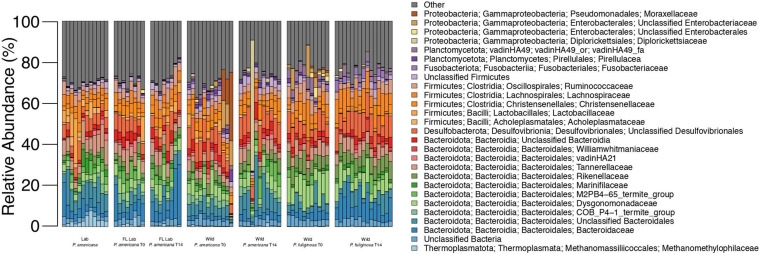
Relative abundance of microbial families across each treatment group. Each bar represents an individual insect gut. All families that represent >5% of sequences from any one sample are listed in the legend; less abundant families are grouped together under other.

16S rRNA gene amplicon sequencing was completed for the hindguts of 90 unique cockroaches across the control and three treatment groups. A total of 6,969,201 sequences were generated, with 4,927,318 remaining after quality filtering and classification (Supplementary Table 1). Across all treatments, hindguts were dominated by members of the Bacteroidota, Firmicutes, and Desulfobacterota phyla. These three phyla comprised 32.84–92.04% of any one individual sample (Figure 1). Members of the Bacteroidota phyla were most abundant across all samples, comprising an average of 41.43% and a median of 42.51% (Figure 1). In contrast, the Firmicutes and Desulfobacterota phyla comprise an average of 30.16 and 10.22% across all samples (Figure 1).

The Bacteroidota observed in the cockroach guts represented a diverse array of families. An average of 2.74% of sequences across all samples belong to unclassified members of the Bacteroidia class with an additional 6.51% of sequences belonging to unclassified members of the Bacteroidales order (Figure 1). Nine other Bacteroidota families were especially abundant across all samples and comprised >5% of the total sequences from at least one sample. These include: Bacteroidaceae, COB P4-1 termite group, Dysgonomonadaceae, M2PB4-65 termite group, Marinifilaceae, Rikenellaceae, Parabacteroides, vadinHA21, and Williamwhitmaniaceae (Figure 1). All of these families except the M2PB4-65 termite group were present within all 90 samples and comprised anywhere from 0.005 to 17.22% of all sequences for any one sample (Figure 1). Of these families, Dysgonomonadaceae and Parabacteroides had the highest average relative abundance across all samples, at 6.11 and 5.37% (Figure 1). Members of the Dysgonomonadaceae and Parabacteroides families are commonly found in the gut microbiome of animals and certain species of Parabacteroides have previously been associated with obesity and inflammation (Wang et al., 2019).

Most of the highly abundant Firmicutes families belonged to the Clostridia class (Figure 1). Members include the Christensenellaceae, Lachnospiraceae, and Ruminococcaceae families which composed an average of 3.38, 5.04, and 3.98% of sequences across all samples (Figure 1). Acholeplasmataceae and Lactobacillaceae, both members of the Bacilli class, were also present with an average relative abundance of 1.08 and 1.51%. Finally, an average of 2.08% of sequences across all samples were unclassifiable members of the Firmicutes phylum (Figure 1).

The majority of the Desulfobacterota present in our experimental samples belonged to unclassified members of the Desulfovibrionales order, which comprised an average of 7.85% of sequences across all samples (Figure 1). Desulfovibrionales are commonly found in the gut microbiome of animals and have been associated with human diseases including obesity and type 2 diabetes (Rinninella et al., 2019). Eight Desulfobacterota families as well as sequences that belong to unclassified members of the Desulfobacterota phylum, Desulfuromonadia class, and the Desulfobacterales and Desulfobulbales orders were also present in select experimental samples at low abundances (Figure 1).

In addition to the three dominant phyla, bacteria from the Proteobacteria phylum was also present within all 90 experimental samples. Four families comprised the majority of these Proteobacteria: Diplorickettsiaceae, unclassified members of the Enterobacterales order, unclassified members of the Enterobacteriacae order, and Moraxellaceae (Figure 1). These families were present at low abundance for most samples, composing an average of 0.38–1.03% (Figure 1). The maximum relative abundance for these families ranged from 5.03 to 62.62% and for each family, the individual sample with the maximum relative abundance belonged to the wild treatment group (Figure 1). Notably, the majority of the Diplorickettsiaceae present in our samples are members of the Rickettsiella genus, which is commonly thought to be an intracellular insect parasite (Leclerque and Kleespies, 2012). Interestingly, most samples contained <0.5% sequences from the Diplorickettsiaceae family (Figure 1). However, eight samples from the wild *P. americana* group had a high (>0.5%) abundance of Diplorickettsiaceae, with a maximum abundance of 63.62% in a wild *P. americana* day 14 sample (Figure 1).

### Wild-Collected Cockroaches Exhibit High Alpha Diversity That Decreases on Being Housed in Laboratory Conditions

**FIGURE 2:**
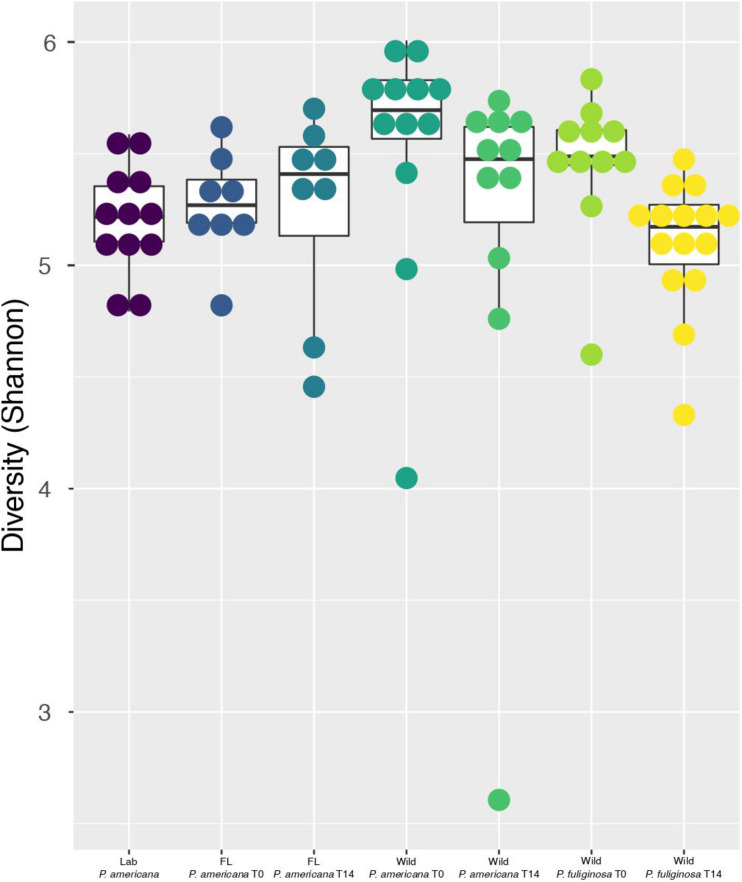
Boxplots show Shannon diversity indices for each group. For each boxplot, the bars delineate the median, the hinges represent the lower and upper quartiles, and the whiskers extend to the lesser of the most extreme value or 1.5 times the interquartile range. Within each boxplot, a dot plot shows the spread of Shannon diversity indices across the group. Before calculating Shannon diversity indices, libraries were resampled to a depth of the sample with the fewest sequences (4082). A full table with all of the paired Wilcoxon rank-sum test with Bonferroni correction and the resulting *p*-values is located in Supplementary Table 1.

Previous work demonstrates that the cockroach hindgut microbiome is highly diverse (Schauer et al., 2012, 2014; Bertino-Grimaldi et al., 2013; Dietrich et al., 2014; Wada-Katsumata et al., 2015; Tinker and Ottesen, 2016, 2020; Renelies-Hamilton et al., 2021). Congruent with this, all cockroach samples, except for one outlier from the day 14 wild *P. americana* treatment group, had a Shannon Diversity metric greater than 4.0 (Figure 2). Pielou’s evenness was also high across all groups at each timepoint, with an average of 0.83 and a median of 0.84 across all samples. Overall, both laboratory populations had similar alpha diversity, but there were significant differences between the laboratory and wild cockroach groups (Figure 2 and Supplementary Table 2). Using the Wilcoxon rank-sum test with Bonferroni correction, we found that wild-collected *P. americana* exhibited significantly higher alpha diversity than both in-house and Florida laboratory *P. americana* populations as well as the wild-collected *P. fuliginosa* (Supplementary Table 2). This alpha diversity decreased on housing in the laboratory, and we found no significant difference (*p* > 0.05) between the in-house laboratory or Florida laboratory *P. americana* and either day 14 wild *P. americana* or *P. fuliginosa* groups (Supplementary Table 2). In addition, we found no significant difference in alpha diversity between the two wild treatment groups at time 0. Interestingly, while there was a significant difference (*p* > 0.05) between the time 0 *P. americana* and the day 14 *P. fuliginosa* groups and vice versa, there was no significant difference in alpha diversity between the two wild-collected groups after 14 days. This suggests that both species lost diversity at similar rates upon housing in the laboratory.

### Hindgut Microbial Communities Cluster by Treatment Group, Host Species, and Sample Origin

**FIGURE 3:**
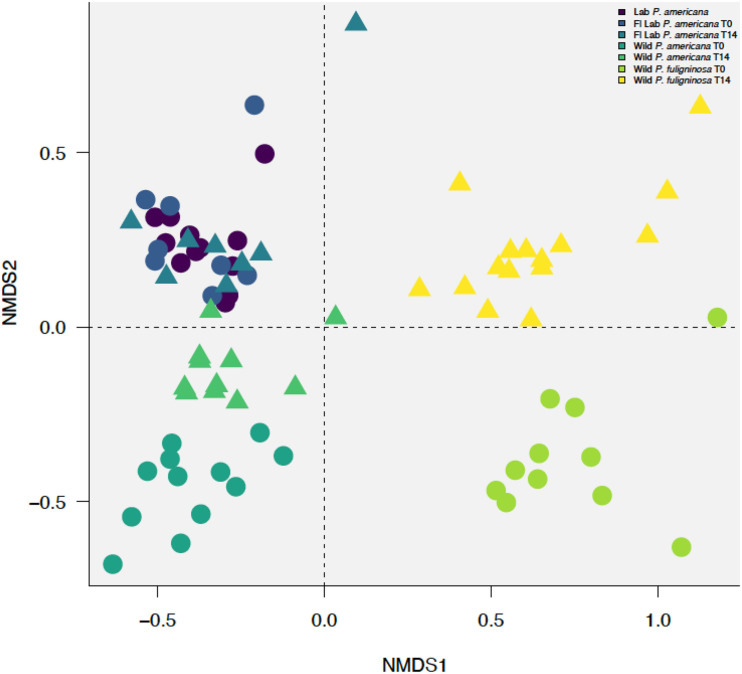
Non-metric multidimensional scaling (NMDS) plot with a stress value of 0.0889 constructed with weighted Bray–Curtis metrics based on the distribution of OTUs (97% sequence identity). Before construction, libraries were resampled to a depth of the sample with the fewest sequences (4082). Clustering by treatment group with (*R* = 0.7349, *p* = 0.001) and without timepoints (*R* = 0.653, *p* = 0.001), host species (*R* = 0.8241, *p* = 0.001), and laboratory or wild origin (*R* = 0.3113, *p* = 0.001) was supported by ANOSIM.

Previous research demonstrates that the cockroach hindgut microbiome is shaped by various compounding biological and environmental factors (Bracke et al., 1978; Bertino-Grimaldi et al., 2013; Dietrich et al., 2014; Schauer et al., 2014; Wada-Katsumata et al., 2015; Tinker and Ottesen, 2020). We used NMDS to visualize Bray–Curtis dissimilarities among our samples (Figure 3 and Supplementary Figure 1). When plotting all samples, we identified a clear separation between the laboratory and wild groups (Figure 3 and Supplementary Figure 1). Furthermore, we identified separation among the wild groups by both species and time point (Figure 3 and Supplementary Figure 1). However, the in-house laboratory and the Florida laboratory group at both timepoints clustered together (Figure 3 and Supplementary Figure 1). When we followed up with ANOSIM, we found there was no statistically significant difference between the in-house and Florida laboratory cockroaches or the two timepoints for the Florida lab cockroaches. In contrast, there was significant separation between the wild *P. americana* (*R* = 0.4825, *p* = 0.001) and wild *P. fuliginosa* (*R* = 0.649, *p* = 0.001) at each timepoint. When the dataset was analyzed as a whole, we found that clustering by treatment group with (*R* = 0.7349, *p* = 0.001) and without timepoints (*R* = 0.653, *p* = 0.001), host species (*R* = 0.8241, *p* = 0.001), and laboratory or wild origin (*R* = 0.3113, *p* = 0.001) was supported by ANOSIM.

### Within- and Between-Group Comparisons Show Differences in Hindgut Microbiome Composition

**FIGURE 4:**
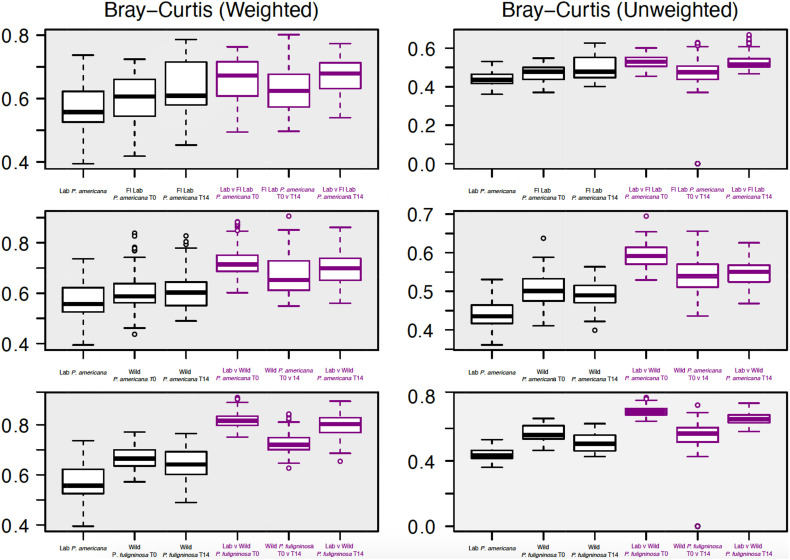
Boxplots show weighted (right) and unweighted (left) pairwise Bray–Curtis dissimilarity metrics among (black) and between (purple) each group. For each boxplot, the bars delineate the median, the hinges represent the lower and upper quartiles, and the whiskers extend to the lesser of the most extreme value or 1.5 times the interquartile range. Before calculating Bray–Curtis dissimilarity metrics, libraries were resampled to a depth of the sample with the fewest sequences (4082). A table with all of the paired Wilcoxon rank-sum tests with Bonferroni correction and the resulting *p*-values is located in Supplementary Table 3.

We were interested in examining differences in within-group variability across populations (Figure 4 and Supplementary Table 3). We found that our in-house laboratory *P. americana* populations showed the lowest within-group variability by both weighted and unweighted Bray–Curtis metrics among the populations tested (Figure 4 and Supplementary Table 3). It was significantly lower than the Florida laboratory *P. americana* at day 14 and the wild *P. fuliginosa* at both time points using weighted measurements (Figure 4 and Supplementary Table 3). When using unweighted measurements, it was significantly lower than all of the treatments (Figure 4 and Supplementary Table 3). Within-group variability was highest for the wild-captured populations, with wild-collected *P. fuliginosa* showing significantly greater variability than wild-collected *P. americana* by weighted measurement (Figure 4 and Supplementary Table 3). Both groups of wild-collected cockroaches showed slight decreases in within-group variability upon housing in the laboratory, but the only significant difference was in unweighted Bray–Curtis comparisons between T0 and T14 wild-captured *P. fuliginosa* (Figure 4 and Supplementary Table 3).

We also compared the between-group dissimilarity for the laboratory *P. americana* control with all other groups (Figure 4). Consistent with ANOSIM results, we found that the between-group dissimilarity for each treatment was significantly higher than the among group dissimilarity (Figure 4). For the Florida laboratory *P. americana*, between-group dissimilarity vs. in-house laboratory cockroaches remained consistent over both time points using both weighted and unweighted metrics. However, we saw a lower between-group dissimilarity vs. our in-house laboratory population after 14 days of laboratory cultivation for both the wild *P. americana* and *P. fuliginosa* groups. Interestingly, there was a greater difference in the unweighted metrics than the weighted metrics, suggesting that the decrease in dissimilarity is likely due to a loss of low-abundance, environmentally associated microbes.

### Differential Abundance Analysis Reveals Unique Taxa Associated With Each Group

**FIGURE 5:**
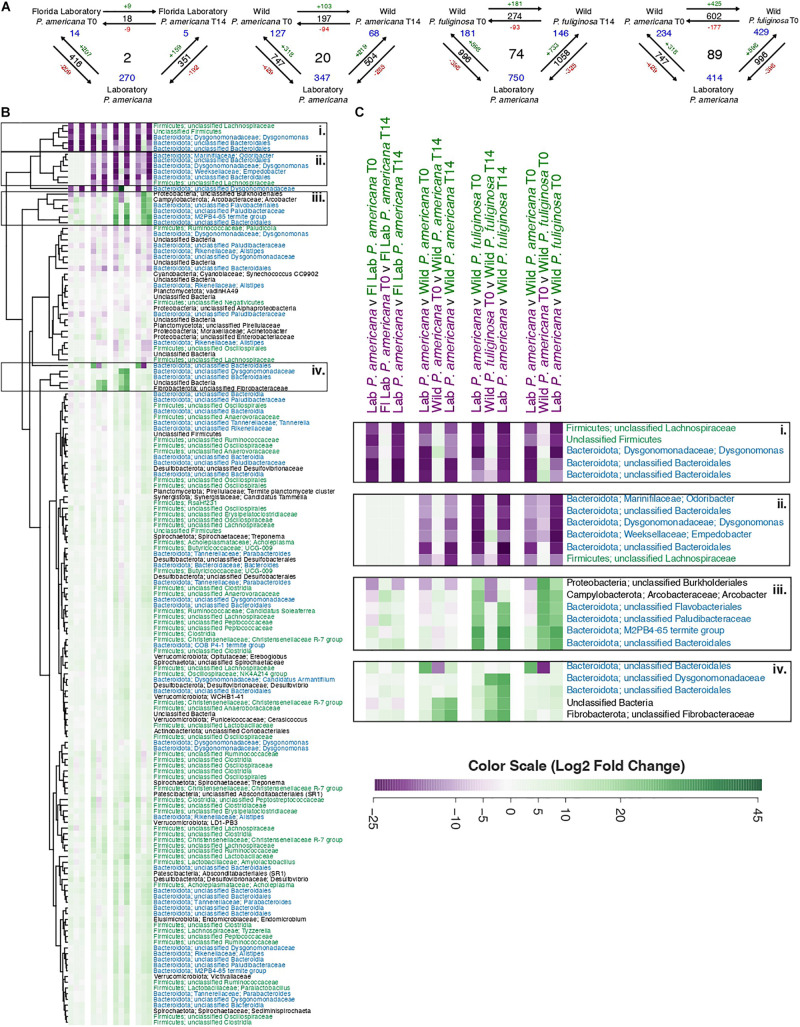
**(A)** Visual representation displaying the number of significant log2-fold changes in abundance between each treatment group. These calculations assumed an adjusted *p*-value of ≤0.05 and were made using the Wald test with Benjamini–Hochberg adjustment as implemented in the DESeq package. Black numbers indicate the total number of statistically significant changes in differential abundance between the two treatment groups. Red (negative) or green (positive) numbers in conjunction with the arrows indicate the direction of the log2-fold change. Finally, blue numbers indicate the number of OTUs that have a significant change in differential abundance across either two groups (corners) or all groups (center). A table containing the raw data from DESeq for the significant log2-fold changes from all pairwise comparisons is located in Supplementary Table 4. **(B)** A heatmap of the 158 OTUs which have significant log2-fold changes across three or more treatment groups. Each row in the heatmap represents a unique OTU, with replicated labels representing multiple OTUs assigned to the same taxonomic group. For each pairwise comparison, the reference is the first pair listed. Each box in the heatmap represents unscaled log2-fold change and ranges from a minimum value of –26.31 (purple) to a maximum of 45.47 (green) with the color scale centered at white. To enable visualization of broader taxonomic patterns, row labels are color coded by phylum assignment, with members of the Bacteroidota phyla in blue, Firmicutes in green, and all others in black. A table containing the raw data and adjusted *p*-values from DESeq2 for these 158 OTUs is located in Supplementary Table 5. **(C)** A close-up of the highlighted sections of the heatmap presented in **B**.

In order to evaluate OTUs driving these between-group differences, we used DESeq2 to identify significantly enriched or depleted OTUs across groups (Supplementary Table 4). We found that there were 416 OTUs with a significant differential abundance between the laboratory *P. americana* and the Florida *P. americana* at time 0, 747 for the laboratory *P. americana* and the wild *P. americana* at time 0, and 996 for the laboratory *P. americana* and the wild *P. fuliginosa* at time 0 (Figure 5A). The number of OTUs with a significant differential abundance between the laboratory *P. americana* decreased to 351 for the Florida *P. americana*, 504 for the wild *P. americana*, and increased to 1,058 for the wild *P. fuliginosa* after 14 days in the laboratory environment (Figure 5A). When we compared the time 0 and day 14 samples within each group, we found that far fewer OTUs had significant compositional differences (Figure 5A). We found 18 between the Florida laboratory *P. americana* at each time point, 197 between the wild *P. americana* at each time point, and 274 between the wild *P. fuliginosa* at each time point (Figure 5A). Overall, this demonstrates that origin (laboratory v. wild) and species strongly impact the cockroach hindgut microbiome but that taxonomic differences are typically reduced when insects are housed in identical conditions.

We found that there were 602 OTUs with a significant differential abundance between the sympatrically collected wild populations of *P. americana* and *P. fuliginosa* (Figure 5A), suggesting that host species results in differences in hindgut microbiome composition among sympatric populations of closely related species. Interestingly, this value was comparable to that found when comparing the laboratory *P. americana* and the wild *P. americana* at time 0 (747) and the laboratory *P. americana* and the wild *P. fuliginosa* at time 0 (996), also confirming an important role for early environment in shaping the hindgut microbiome.

Figure 5B shows a heatmap illustrating OTUs that exhibit significant log2-fold changes across our four sets of three-way comparisons (Supplementary Table 5). We found 2, 20, and 74 OTUs with significant log2-fold changes across all three paired comparisons for the Florida *P. americana*, wild *P. americana*, and wild *P. fuliginosa* treatment groups, respectively (Figure 5A). We also found 89 OTUs with significant log2-fold changes found in the comparison of laboratory *P. americana* with the two wild-collected populations (Figure 5A). Removal of redundant sets resulted in a list of 158 OTUs which have exhibited significant log2-fold changes across all three pairwise comparisons of 1 or more treatment groups (Supplementary Table 5). When we visualize these 158 OTUs in an unscaled heatmap (Figure 5B), there are two notable trends. First, the majority of these OTUs have low taxonomic resolution and second, the majority of these OTUs have statistically significant, but minimal log2-fold changes (Figure 5B and Supplementary Table 5). Within the heatmap, there were 22 OTUs that had a large change across multiple treatment groups (Figure 5B). When we explicitly examine these OTUs they fall into four general categories (Figure 5C): those (1) associated only with the laboratory control *P. americana*; (2) associated with both the laboratory control *P. americana* and the Florida laboratory *P. ameriana*; (3) associated with wild *P. fuliginosa*; and (4) associated with wild *P. americana*. Fifteen out of 22 (and 57 out of the full 158) of these OTUs represent diverse Bacteroidota, including two *Dysgonomonas* genus OTUs as well as multiple OTUs that could not be classified beyond the order or family level. Many of the Bacteroidota genera and families include multiple OTUs showing contrasting patterns in abundance across groups. A number of Firmicutes also show group-specific patterns, although they were more highly represented among organisms showing smaller fold changes between groups. As these groups are thought to play diverse roles in carbohydrate metabolism (Vera-Ponce de León et al., 2020), further studies are required to determine the functional implications of these OTU shifts. Another interesting taxon is *Arcobacter*, which was associated with wild *P. fuliginosa* and is thought to be an emerging pathogen (Collado and Figueras, 2011; Barboza et al., 2017).

## Discussion

The cockroach is emerging as a common model organism for studying the gut microbiome due to their rich hindgut microbial community, omnivorous diet, and gut structure, which is analogous to the human stomach. Our goal was to better understand the factors that drive assembly and biodiversity in the cockroach gut microbiome. Previous work on the cockroach gut microbiome demonstrates that cockroaches have a gut microbiome which is more typical of omnivorous mammals than insects outside of the Blattodea order (Schauer et al., 2012; Tinker and Ottesen, 2016, 2020). Much like the mammalian gut microbiome, the cockroach gut microbiome is highly complex and primarily composed of bacterial lineages from the Bacteroidota and Firmicutes phyla (Schauer et al., 2012; Tinker and Ottesen, 2016, 2020). Our work was congruent with this and showed that all cockroaches contained a high abundance of uncharacterized, insect-associated microbial families.

Previous work showed that host species shapes the gut microbiome of cockroaches (Tinker and Ottesen, 2020). Consistent with this, we observed that sympatric populations of wild-collected cockroaches belonging to different species showed significantly different hindgut microbiome compositions, with over 602 microbial OTUs differing significantly in abundance between these two species. Upon housing in identical laboratory conditions, the wild-collected populations showed decreases in alpha and beta diversity. Wild-caught *Drosophila* gut microbiomes also decrease in diversity when brought into a laboratory setting (Martinson et al., 2017). This is interesting, as the *Drosophila* gut microbiome appears to be governed by different rules of assemblage, with local sampling environment shaping the gut microbiome rather than host species (Martinson et al., 2017). Renelies-Hamilton et al. (2021) have suggested that co-housing can, to some extent, overcome founder’s effects in gut microbiome composition in cockroaches (Martinson et al., 2017). Therefore, it would be interesting to observe the extent to which these differences could be resolved by co-housing with laboratory-origin populations and/or rearing in the laboratory environment.

Interestingly, a comparable number of OTUs (747) were observed to exhibit significantly different abundances between laboratory and wild-caught *P. americana* as were differentially abundant between the sympatric wild populations of *P. americana* and *P. fuliginosa* (602). This is congruent with previous studies that have included wild-captured cockroaches (Bertino-Grimaldi et al., 2013; Pérez-Cobas et al., 2015; Tinker and Ottesen, 2016; Kakumanu et al., 2018), which have also typically found a distinct and more diverse gut microbiome. Housing in the laboratory resulted in decreases in alpha diversity, decreases in within-group beta diversity, and a significant change in abundance for 197 OTUs. However, the laboratory-housed *P. americana* of wild origin retained a distinct hindgut microbiome from the laboratory-raised *P. americana*, with 504 OTUs exhibiting significant differences between these two populations. Notably, 347 of these OTUs were also significantly different between the laboratory cockroaches and day 0 wild-captured cockroaches.

We found substantially greater similaritiesbetween the UGA and Florida laboratory populations of *P. americana*. In general, while our in-house laboratory populations of cockroach showed greater within-group similarity than the Florida-sourced laboratory population (potentially as a result of disturbance resulting from the transport and altered housing of Florida-sourced cockroaches), both showed low within-group variability and similar levels of alpha diversity. The two laboratory populations clustered together to the exclusion of both wild-origin populations in our NMDS plot, although 270 OTUs were found to be significantly different between our in-house laboratory populations and both day 0 and day 14 Florida laboratory cockroaches. Interestingly, unlike the wild-captured cockroaches, the Florida-origin laboratory population showed significant changes in the abundance of only 18 microbial OTUs upon housing in our laboratory, suggesting substantially smaller shifts in microbiome composition upon transfer from one laboratory to another than that occurring during the transition to the wild to loratory conditions.

We also found it notable that many of the group-specific OTUs belonged to the Bacteroidota phylum. Across all groups, 57 of the 158 significant OTUs, or 36.08%, were members of the Bacteroidota phylum. In contrast, Bacteroidota only represents 20.53% of the total number of OTUs. This increase in representation suggests that Bacteroidota strains may be particularly likely to vary across populations, perhaps since many are strict anaerobes without the ability to make endospores and therefore could be less easily transmitted through/acquired from the environment. Alternatively, they may be particularly responsive to dietary or environmental differences between groups.

We also found it notable that Rickettsiella and Arcobacter (Figure 5 and Supplementary Tables 4, 5) were highly abundant in wild *P. americana* and wild *P. fuliginosa*, respectively. Rickettsiella are most commonly known as intracellular insect pathogens (Leclerque and Kleespies, 2012), however, there is one symbiotic species found in aphids that can be found in extracellular tissues or in the hemolymph (Tsuchida et al., 2010). Rickettsiella can be transmitted both vertically and horizontally between insects (Iasur-Kruh et al., 2013; Marshall et al., 2017), and certain Rickettsiella species have been found to be transferred from insects to mammals via insect bites (Anstead and Chilton, 2014). Similarly, Arcobacter is an emerging animal and human pathogen with multiple roles of transmission (Collado and Figueras, 2011; Barboza et al., 2017). As cockroaches have previously been thought to act as a reservoir for pathogenic microbes (Pai, 2013; Menasria et al., 2014; Memona et al., 2016; Moges et al., 2016), it is unclear whether the insects harboring these taxa were in diseased states or acting as mechanical vectors. However, these findings highlight the necessity of future work focused on the role of cockroaches in disease transmission.

This work provides new insight into the impact of prolonged cultivation in the laboratory on the gut microbiome of cockroaches. We found that culture in the laboratory consistently decreased hindgut microbiome diversity, and that laboratory-reared and wild-captured cockroaches belonging to the same species had distinct hindgut microbial communities, while long-separated laboratory populations of the same species were more similar in composition overall. Further, laboratory-reared populations showed smaller shifts in hindgut microbial community composition upon transfer to a new laboratory than wild-captured cockroaches did upon transfer to the same laboratory. Interestingly, differences between the hindgut microbial community of laboratory-reared and wild-caught cockroaches were similar in scale to the differences between sympatric populations of wild-captured cockroaches from different species, but while laboratory cultivation resulted in similar losses of microbial diversity between wild-captured populations of different species it did not cause them to become substantially more similar overall. Together, these illustrate strong effects of host biology, early environment, and recent environment on the gut microbiome of cockroaches, and underscores the potential of the cockroach as a model system for untangling the modes of action and interactions between these drivers in shaping host–microbiome interactions.

## Data Availability Statement

The 16S rRNA sequences generated from this experiment were submitted to the NCBI Sequence Read Archive and are available under the accession numbers SRP075213, PRJNA726249, SRX1763652, and SRP132948. Insect host sequences were deposited in GenBank under accession numbers MH360270 and MH360286.

## Author Contributions

KT collected and processed the samples, prepared the sequencing libraries, analyzed the data, and wrote the manuscript. EO procured the funding, assisted in data analysis, and edited the manuscript. Both authors contributed to the article and approved the submitted version.

## Conflict of Interest

The authors declare that the research was conducted in the absence of any commercial or financial relationships that could be construed as a potential conflict of interest.

## Publisher’s Note

All claims expressed in this article are solely those of the authors and do not necessarily represent those of their affiliated organizations, or those of the publisher, the editors and the reviewers. Any product that may be evaluated in this article, or claim that may be made by its manufacturer, is not guaranteed or endorsed by the publisher.
