# Supplementary material for: Differences in Gut Microbiome Composition Between Sympatric Wild and Allopatric Laboratory Populations of Omnivorous Cockroaches
Source: Front Microbiol. 2021 Jul 28;12:703785. doi: 10.3389/fmicb.2021.703785 (PMC8355983; doi:10.3389/fmicb.2021.703785)
Supplement: Supplementary file 1 [file Image_1.PDF]

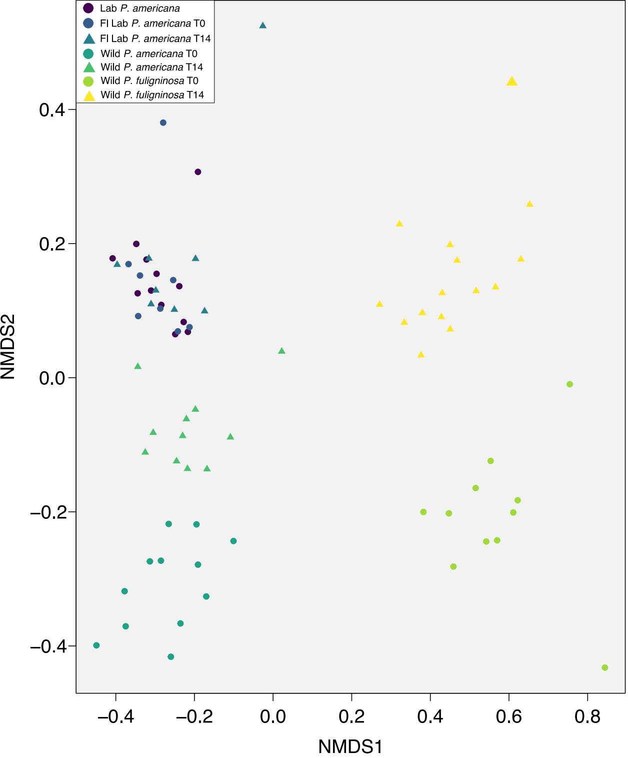

SI Figure 1. Nonmetric multidimensional scaling (NMDS) plot with a stress value of 0.0813 constructed with unweighted Bray-Curtis metrics based on the distribution of OTUs (97% sequence identity). Before construction, libraries were resampled to a depth of the sample with the fewest sequences (4112).
