# Supplementary material for: Differences in Gut Microbiome Composition Between Sympatric Wild and Allopatric Laboratory Populations of Omnivorous Cockroaches
Source: Front Microbiol. 2021 Jul 28;12:703785. doi: 10.3389/fmicb.2021.703785 (PMC8355983; doi:10.3389/fmicb.2021.703785)
Supplement: Supplementary file 3 [file Table_2.DOCX]

| **Pairwise Comparison** | **p-value** |
| --- | --- |
| Lab *P. americana* v Fl Lab  *P. americana* T0 | 0.401 |
| Lab *P. americana* v Fl Lab *P. americana* T14 | 0.357 |
| Lab *P. americana* v Wild *P. americana* T0 | 0.015* |
| Lab *P. americana* v Wild *P. americana* T14 | 0.098 |
| Lab *P. americana* v Wild *P. fuligninosa* T0 | 0.012* |
| Lab *P. americana* v Wild *P. fuligninosa* T14 | 0.621 |
| Fl Lab  *P. americana* T0 v Fl Lab *P. americana* T14 | 0.621 |
| Fl Lab  *P. americana* T0 v Wild *P. americana* T0 | 0.033* |
| Fl Lab  *P. americana* T0 v Wild *P. americana* T14 | 0.225 |
| Fl Lab  *P. americana* T0 v Wild *P. fuligninosa* T0 | 0.015* |
| Fl Lab  *P. americana* T0 v Wild *P. fuligninosa* T14 | 0.667 |
| Fl Lab *P. americana* T14 v Wild *P. americana* T0 | 0.134 |
| Fl Lab *P. americana* T14 v Wild *P. americana* T14 | 0.778 |
| Fl Lab *P. americana* T14 v Wild *P. fuligninosa* T0 | 0.357 |
| Fl Lab *P. americana* T14 v Wild *P. fuligninosa* T14 | 0.371 |
| Wild *P. americana* T0 v Wild *P. americana* T14 | 0.166 |
| Wild *P. americana* T0 v Wild *P. fuligninosa* T0 | 0.173 |
| Wild *P. americana* T0 v Wild *P. fuligninosa* T14 | 0.012* |
| Wild *P. americana* T14 v Wild *P. fuligninosa* T0 | 0.621 |
| Wild *P. americana* T14 v Wild *P. fuligninosa* T14 | 0.098 |
| Wild *P. fuligninosa* T0 v Wild *P. fuligninosa* T14 | 0.012* |

**SI Table 2. Wilcoxon Rank Test with Bonferroni Correction for Shannon Diversity Metrics**

*p-value <0.05
