# Supplementary material for: Differences in Gut Microbiome Composition Between Sympatric Wild and Allopatric Laboratory Populations of Omnivorous Cockroaches
Source: Front Microbiol. 2021 Jul 28;12:703785. doi: 10.3389/fmicb.2021.703785 (PMC8355983; doi:10.3389/fmicb.2021.703785)
Supplement: Supplementary file 4 [file Table_3.docx]

**SI Table 3. Wilcoxon Rank Test with Bonferroni Correction for Bray-Curtis Metrics**

| **Weighted** | |
| --- | --- |
| **Pairwise Comparison** | **p-value** |
| Lab *P. americana* v Fl Lab  *P. americana* T0 | 0.117 |
| Lab *P. americana* v Fl Lab *P. americana* T14 | 0.0028* |
| Lab *P. americana* v Wild *P. americana* T0 | 0.043* |
| Lab *P. americana* v Wild *P. americana* T14 | 0.011* |
| Lab *P. americana* v Wild *P. fuligninosa* T0 | 5.5e-09* |
| Lab *P. americana* v Wild *P. fuligninosa* T14 | 1.7e-07* |
| Fl Lab  *P. americana* T0 v Fl Lab *P. americana* T14 | 0.235 |
| Fl Lab  *P. americana* T0 v Wild *P. americana* T0 | 0.840 |
| Fl Lab  *P. americana* T0 v Wild *P. americana* T14 | 0.644 |
| Fl Lab  *P. americana* T0 v Wild *P. fuligninosa* T0 | 0.002* |
| Fl Lab  *P. americana* T0 v Wild *P. fuligninosa* T14 | 0.043* |
| Fl Lab *P. americana* T14 v Wild *P. americana* T0 | 0.126 |
| Fl Lab *P. americana* T14 v Wild *P. americana* T14 | 0.410 |
| Fl Lab *P. americana* T14 v Wild *P. fuligninosa* T0 | 0.032* |
| Fl Lab *P. americana* T14 v Wild *P. fuligninosa* T14 | 0.410 |
| Wild *P. americana* T0 v Wild *P. americana* T14 | 0.410 |
| Wild *P. americana* T0 v Wild *P. fuligninosa* T0 | 1.1e-06* |
| Wild *P. americana* T0 v Wild *P. fuligninosa* T14 | 7.7e0-4* |
| Wild *P. americana* T14 v Wild *P. fuligninosa* T0 | 2.5e0-4* |
| Wild *P. americana* T14 v Wild *P. fuligninosa* T14 | 0.032* |
| Wild *P. fuligninosa* T0 v Wild *P. fuligninosa* T14 | 0.058 |
| **Unweighted** | |
| **Pairwise Comparison** | **p-value** |
| Lab *P. americana* v Fl Lab  *P. americana* T0 | 0.003* |
| Lab *P. americana* v Fl Lab *P. americana* T14 | 6.1e-06* |
| Lab *P. americana* v Wild *P. americana* T0 | 1.6e-15* |
| Lab *P. americana* v Wild *P. americana* T14 | 3.5e-11* |
| Lab *P. americana* v Wild *P. fuligninosa* T0 | < 2e-16* |
| Lab *P. americana* v Wild *P. fuligninosa* T14 | 1.7e-15* |
| Fl Lab  *P. americana* T0 v Fl Lab *P. americana* T14 | 0.227 |
| Fl Lab  *P. americana* T0 v Wild *P. americana* T0 | 0.004* |
| Fl Lab  *P. americana* T0 v Wild *P. americana* T14 | 0.092* |
| Fl Lab  *P. americana* T0 v Wild *P. fuligninosa* T0 | 1.8e-08* |
| Fl Lab  *P. americana* T0 v Wild *P. fuligninosa* T14 | 0.005* |
| Fl Lab *P. americana* T14 v Wild *P. americana* T0 | 0.205 |
| Fl Lab *P. americana* T14 v Wild *P. americana* T14 | 0.886 |
| Fl Lab *P. americana* T14 v Wild *P. fuligninosa* T0 | 8.5e-06* |
| Fl Lab *P. americana* T14 v Wild *P. fuligninosa* T14 | 0.318 |
| Wild *P. americana* T0 v Wild *P. americana* T14 | 0.092 |
| Wild *P. americana* T0 v Wild *P. fuligninosa* T0 | 7.9e-09* |
| Wild *P. americana* T0 v Wild *P. fuligninosa* T14 | 0.920 |
| Wild *P. americana* T14 v Wild *P. fuligninosa* T0 | 9.4e-11* |
| Wild *P. americana* T14 v Wild *P. fuligninosa* T14 | 0.150 |
| Wild *P. fuligninosa* T0 v Wild *P. fuligninosa* T14 | 1.8e-08* |

*p-value <0.05
